# School‐based yoga and mindfulness interventions for young adolescents: A qualitative study in a disadvantaged area

**DOI:** 10.1111/bjhp.12793

**Published:** 2025-03-18

**Authors:** Amy L. Sumner, Tina Cartwright, Haiko Ballieux, Trudi Edginton

**Affiliations:** ^1^ University of Westminster London UK; ^2^ City University of London London UK

**Keywords:** adolescents, low socioeconomic status, mindfulness, qualitative research, school‐based interventions, well‐being, yoga

## Abstract

**Objectives:**

With raising rates of mental health problems, mind–body interventions are increasingly being integrated in schools to support children and adolescents' mental health and well‐being. The aim of this study was to explore young adolescents' experiences of yoga and mindfulness and the acceptability of delivery within the school curriculum in an area of high deprivation.

**Design:**

Qualitative group interviews with young adolescents embedded within a larger feasibility study exploring the universal (class‐wide) delivery of yoga and mindfulness interventions.

**Methods:**

After participation in separate 10‐week yoga or mindfulness interventions, 45 adolescents (12–13 years old; 66.7% male) took part in semi‐structured group interviews exploring perceptions, acceptability, and experiences of each intervention. Data was analysed using inductive thematic analysis.

**Results:**

Two overarching themes were identified, evident across both mindfulness and yoga groups. “Facilitators and barriers to engagement” outlined key factors impacting acceptability, including prior perceptions. Teacher qualities of non‐reactivity and respect, an invitational approach to teaching, and interactive sessions were highly valued. Secondly, participants described a range of “psychosocial impacts”, including increased emotional regulation, positive mindset and self‐confidence, and greater focus and concentration. Physical benefits were also reported in the yoga group.

**Conclusion:**

This is the first study to explore the acceptability and impact of universal yoga and mindfulness interventions with an ethnically diverse sample of disadvantaged young adolescents in the United Kingdom. The findings suggest mind–body interventions can help children and adolescents develop skills to better manage challenges in their everyday lives, but they require further integration into the curriculum for optimal benefit.

**Statement of contribution**

**What is already known on this subject?**

- Mental health problems affect up to 14% of adolescents, with higher rates experienced by those from more deprived areas.
- Evidence supports the benefits of yoga and mindfulness interventions on adolescents' well‐being and socio‐emotional skills.
- However, the implementation and effectiveness of school‐based universal delivery of mind–body interventions are mixed.

**What does this study add?**

- Factors impacting engagement with yoga and mindfulness in low SES ethnic minority adolescents.
- Biopsychosocial benefits of emotional regulation, positivity, confidence, strength, and focus.
- Potential of universal interventions to teach socio‐emotional skills to navigate life challenges.

## Introduction

There is growing acceptance that mental health is just as important as physical health for children to grow into healthy adults. The period of childhood offers an important window in which to develop socio‐emotional skills and coping resources to manage stressors and support well‐being during and beyond adolescence (Cheetham‐Blake et al., 2019). Whilst there is increasing interest in understanding the factors associated with optimal psychological functioning or 'flourishing' for children and adolescents (Huppert & So, 2013; Witten et al., 2019), the rising rates of mental health problems internationally have emerged as the primary focus (Hafstad & Augusti, 2021). A recent large‐scale survey suggests that over 20% of 8–16‐year‐olds in England have a probable mental disorder (Newlove‐Delgado et al., 2023). Given the negative outcomes associated with childhood mental ill‐health, including poorer educational achievement, lower school engagement (Goodman et al., 2011), increased health risk behaviours (Sadler et al., 2018), and continuing mental health problems in adulthood (Clark et al., 2007; Colman et al., 2007), there is a strong need to develop programmes for children and adolescents that promote self‐regulation, resilience, and well‐being (Kuyken et al., 2017; Sawyer et al., 2012).

To promote optimal well‐being in young people, a biopsychosocial approach is essential, given the established evidence that certain psychosocial risk factors increase the likelihood of developing mental health problems. Unmanaged stress is a significant mediating factor between low socioeconomic status (SES) and adolescent mental health (Devenish et al., 2017; Roberts et al., 2009). More specifically, adolescents from low SES neighbourhoods were found to be 2–3 times more likely to develop mental health problems in comparison with higher SES neighbourhoods (Reiss, 2013) and experience further disadvantage over the life course (McAnally et al., 2021). In contrast, flourishing and resilience may serve as possible mediators between adverse early experiences and health issues in adulthood (Witten et al., 2019). Given the clear relationship between low SES and poor mental health, Devenish et al. (2017) called for interventions that support children and adolescents from low SES neighbourhoods to manage the additional stressors they may face and develop their social, cognitive, and emotional functioning. School‐based interventions thus offer a prime opportunity to promote mental health and socio‐emotional well‐being given their central role in children and adolescents' development (Durlak et al., 2011).

Yoga and mindfulness interventions have grown in popularity in recent years and have shown promising findings for children and adolescents in the school context (Feagans Gould et al., 2016), although research is more developed in adult populations. Mindfulness is commonly defined as “paying attention in a particular way: on purpose, in the present moment, and non‐judgmentally” (Kabat‐Zinn, 1994 p. 4). It invites individuals to focus on present moment sensations, through redirection of attention to the present, and encourages a non‐judgemental and compassionate attitude towards difficult thoughts and emotions, promoting self‐regulation (Segal et al., 2002). Mindfulness can be practiced on its own or can incorporate mindful movement, which has its origins in yoga. Yoga is a holistic system of practices that incorporates mindfulness and meditation together with postures (asanas), breath control (pranayama), and relaxation, to engage both the mind and the body (Case‐Smith et al., 2010). Physical postures promote flexibility and physical strength; breathing exercises improve respiratory functioning; relaxation techniques help to release physical and mental tension; and meditation enhances mind–body awareness and improves attention and emotional regulation skills (Butzer et al., 2016). Importantly, both interventions operate from an invitational stance, encouraging participants to engage in a way that best supports their aims and aspirations (Crane et al., 2017).

Both yoga and mindfulness interventions have shown measurable benefits for children and adolescents' socio‐emotional well‐being, mitigating physical stress responses and facilitating resilience and emotional regulation (e.g. Bergen‐Cico et al., 2015; Daly et al., 2015; Huppert & Johnson, 2010; Kuyken et al., 2013; Miller et al., 2020). In comparison with the quantitative evidence, there is limited qualitative research with children and adolescents to elicit their perceptions and experiences of yoga and mindfulness in schools. US‐based studies have reported a range of socio‐emotional benefits after participation in yoga interventions, including improved emotional and behavioural self‐regulation, stress, self‐esteem, social interaction, relaxation, and sleep (Butzer et al., 2017; Case‐Smith et al., 2010; Conboy et al., 2013; Wang & Hagins, 2015). Similarly, a thematic synthesis of mindfulness interventions described benefits for adolescents' emotional awareness, emotional regulation, stress reduction, coping, social skills, and relaxation (Sapthiang et al., 2019).

Despite evidence suggesting positive effects on well‐being, the majority of studies to date have been conducted in the United States. This is particularly salient in light of the most recent Mental State of the World Report, in which the United Kingdom ranks second to last in terms of average mental health quotient score and last in terms of distress and struggling (Thiagarajan & Newson, 2024), suggesting a specific need for UK‐based interventions to support mental health and well‐being. Previous research has also targeted children and adolescents with a particular need, such as mental health problems, poor academic achievement, or behavioural issues (Bannirchelvam et al., 2017; Case‐Smith et al., 2010; McGeechan et al., 2019; Wisner, 2014). In contrast to more targeted approaches, school‐based universal interventions provide all individuals with the skills to better manage their social, cognitive, and emotional functioning, irrespective of perceived need (Dray et al., 2017). Universal delivery methods may increase the social acceptance of interventions as individuals are not singled out as having a pre‐identified need (Gronholm et al., 2018), in addition to being more cost‐effective (Greenberg & Abenavoli, 2017). Moreover, universal approaches are consistent with the UK policy landscape, which has advocated for whole school approaches to promote the well‐being and resilience of children and adolescents (Greenberg & Abenavoli, 2017; NHS England & Department of Health, 2015).

However, the implementation of school‐based universal approaches is not without challenges, both in terms of engagement and effectiveness (Wilde et al., 2019). A recent fully powered trial in the United Kingdom (MYRIAD) exploring mindfulness (using the same programme as the current study) found no superiority over standard Personal, Social, Health and Economic (PSHE) teaching (Kuyken et al., 2022). They also reported substantial variation in levels of responsiveness amongst pupils, with only half rating it positively (Montero‐Marin et al., 2023). Consequently, further research is needed to understand children and adolescents' perceptions and experiences of school‐based yoga and mindfulness interventions, delivered in a universal way. Indeed, research has highlighted the importance of qualitative studies to better inform the implementation of well‐being programmes (McKeering & Hwang, 2019).

The current study thus explored young adolescents' experiences of two separate universally delivered school‐based programmes, yoga and mindfulness, in a diverse, low SES urban area in the United Kingdom. Using a qualitative approach, the study aimed to explore (a) perceptions of yoga and mindfulness and the acceptability of delivery within the school curriculum, and (b) experiences and perceived effects of the 10‐week programmes.

## Method

### Design

A qualitative approach was adopted to offer in‐depth insight into participants' thoughts, feelings, and perspectives (Braun & Clarke, 2019), underpinned by a critical realist epistemology (Scott, 2007). Qualitative research is especially important in this context, recognizing that young people are the best sources of information about their perceptions and experiences (Vasques et al., 2014). Semi‐structured group interviews were conducted, where the researcher plays a more prominent role than in focus groups, interacting with each participant and checking the consensus with the other members of the group (Brown & Edmunds, 2011). Group interviews were adopted to reduce social desirability bias due to unequal power dynamics between adolescent participants and adult researchers (Kutrovátz, 2017).

This qualitative study forms part of a larger mixed methods feasibility study exploring the impact of school‐based yoga and mindfulness interventions (across 2 year eight cohorts). Participant feedback in Year One was integrated in Year Two to adapt the intervention content and delivery, for example, reducing intervention class size. The research was approved by the University Research Ethics Committee (ETH1718‐1686; ETH1819‐2012). The school was situated in the top 20% of the most deprived areas in England (Ministry of Housing Communities and Local Government 2019).

### Participants and recruitment

Purposeful sampling was adopted after programme completion; teachers were asked to recruit pupils for the interview who varied on a number of factors (gender, engagement with the intervention, and perceived enjoyment) to increase the heterogeneity of the sample. Of the 242 young adolescents who took part in the wider intervention study, 45 participants (12–13 year‐olds; 66.7% male) were approached and consented to take part in a group interview (see Table 1). The interview sub‐sample was reflective of the wider intervention sample in terms of ethnicity (Asian: 51%, Black: 32%, Mixed Ethnicity: 8%, White: 5%, Other: 4%).

**TABLE 1:** Overview of sample.

	Condition			
	Yoga		Mindfulness	
	*N*	%	*N*	%
*Sex*				
Male	14	66.7	16	66.7
Female	7	33.3	8	33.3

### Procedure

Prior to the interview, all parents provided passive consent and participants provided informed consent. Fourteen face‐to‐face group interviews were conducted 1 week after the final class by AS, a female Ph.D. student with extensive prior experience of interviewing children and adolescents. The researcher had no relationship with participants prior to the interviews. All interviews took place in a classroom on the school premises, during lesson time, and lasted 30–45 minutes (average: 35 minutes). A semi‐structured interview guide (Data S1) was developed to facilitate conversation and explore young adolescents' views of the interventions, including classroom experiences (e.g. “What was it like doing yoga/mindfulness with your class?”), any perceived impact (e.g. “Have you noticed any changes in your life?”, “What difficulties, if any, have you experienced?”), and suggestions for improvement. Participants were encouraged to be open and honest in their responses to help adapt future classes.

### Interventions

Separate yoga and mindfulness interventions were delivered to Year 8 pupils for 10 weeks during the 50‐min PSHE lesson.

#### Yoga4Schools

The Yoga4Schools curriculum was designed in partnership with the Teen Yoga Foundation for use with adolescents aged 11–16 years, focused on equipping adolescents with self‐management tools and promoting positive development (Martinus, 2018). The sessions consisted of a short check‐in with participants, a discussion of the weekly theme, various asanas with associated breathing practices, and a final relaxation (see Data S2). The yoga teachers had advanced training with the Teen Yoga Foundation and had extensive experience of working with adolescents. To optimize efficiency and accommodate pupil preferences, participants wore school uniform for the yoga sessions, with appropriate modifications.

#### Mindfulness in Schools Project

The Mindfulness in Schools Project's (MiSP) .b (dot‐be) psycho‐educational curriculum was designed for pupils aged 11–18 years. In each session, new themes were introduced, and skills were taught in a practical way with application to everyday life (see Data S3). The curriculum was based on the principles of MBSR (Kabat‐Zinn, 1982) and was designed to be consistent with effective school‐based interventions for well‐being promotion (Kuyken et al., 2013). The mindfulness teachers were trained in the curriculum by MiSP and were experienced in working with adolescents.

### Data analysis

All interviews were recorded, transcribed verbatim, and anonymised before analysis using NVivo (Version 12). Given the exploratory aims, an inductive approach was taken, where the themes were strongly linked to the content of the data and not preconceived (Patton, 1990). There was considerable overlap between the narratives of yoga and mindfulness participants, which prompted the analysis of interviews as a single corpus whilst remaining primed to the differences between the two interventions.

Descriptive thematic analysis using Braun and Clarke's (2006) six‐step framework was employed to analyse the data, with a semantic approach to theme development, focusing on what participants said and deriving meaning from the explicit views and experiences they voiced. This approach was consistent with the study's realist epistemological underpinnings, where motivations, experiences, and meanings were analysed and interpreted in an unassuming manner, without theorizing the socio‐cultural contexts essential in constructivist approaches (Braun & Clarke, 2006).

The researcher (AS) immersed themselves in the data by transcribing, reading, and re‐reading transcripts to identify themes and patterns in the data. Initial codes were generated to highlight key ideas and concepts in the data. These were categorized into preliminary themes consisting of clusters of related codes, which were then reviewed to ensure coherence and consistency within and across the data set.

To increase rigour, an experienced qualitative researcher (TC) reviewed four transcripts (29%) in the initial stages of analysis to discuss and agree coding and initial thematic structure. Regular analysis meetings were also conducted with the research team to discuss and debate emerging themes and interpretations; any instances of disagreement were discussed until a consensus was reached. The researchers acknowledge that they were active agents in the data analysis process. Therefore, in order to increase transparency, the COREQ criteria for qualitative research guided the methodology and reporting (Tong et al., 2007).

## Results

Two overarching themes were identified relating to young adolescents' perceptions and experiences of the two programmes (See Table 2). Representative quotes are presented throughout the findings (see Data S4 for further illustrative quotes for each theme).^1^

**TABLE 2:** Overview of themes.

Theme	Description
Facilitators and barriers to engagement	**Expectations** Prior perceptions and assumptions about both interventions were varied and could serve as either a barrier or facilitator to initial engagement
	**Teacher qualities** Adolescents highlighted the qualities of nonreactivity, care, and respect as facilitating their engagement with both interventions. However, examples of reactivity (e.g. shouting) served as negative reinforcement, more closely aligned with the traditional class experience
	**Agency** The invitational approach of teaching within both interventions enabled participants to feel more in control and facilitated informed decisions about engagement. However, this could increase disruptive behaviour and negatively impact on engagement
	**Interactivity** Interactive and varied activities within intervention sessions facilitated adolescents' engagement and enjoyment, with a strong preference for active classroom activities. In contrast, lack of activities or perceived boredom hindered engagement
Psychosocial impacts	**Regulating emotions and calming the mind** Young adolescents described improved knowledge of strategies to better regulate their emotions, which helped them to feel calmer and manage stress more effectively
	**Positive mindset and confidence** Participants described a range of positive mental states, increases in self‐confidence, and shifts in their social relationships
	**Focus and concentration** Increases in focus and concentration were felt to contribute to more conducive learning environments
	**Physical performance** Yoga participants, in particular, described changes to their physical health and athletic performance

### Facilitators and barriers to engagement

#### Expectations

The majority of participants had very limited or no prior knowledge of yoga and mindfulness. This could be an enabler or barrier to openness to engage, and there were differences in perceptions about the two practices. Media portrayals particularly influenced views about yoga; for example, as female dominated or for “flexible” (Y2‐F) people. This contrasted with positive perceptions arising from validation from athletes or celebrities: “if they do it then it must have a good benefit” (Y4‐M). Yoga as a physical practice was attractive to several participants: “I got very excited because I wanted to stretch my body” (Y5‐M). In contrast, participants frequently felt that mindfulness was “going to be boring” (M6‐F); however, this was not necessarily borne out in practice. Others felt excited about the opportunity to try something new and learn “to train our minds” (M8‐F) through mindfulness or were open to “give it a try” (M1‐M). For this group of young adolescents, prior expectations of intervention sessions set the scene for their initial engagement and their openness to new experiences.

#### Teacher qualities

The non‐reactivity of intervention teachers was highly valued and described by most participants as key to facilitating their enjoyment of classes and continued engagement. This was exemplified in teachers' reluctance to raise their voices and ability to maintain a calm attitude irrespective of classroom behaviour:

I think [Name] was a nice teacher… she wouldn't shout at us or scream or say ‘I'm done with you’ or ‘you step outside’. She would understand that some kids misbehave, and she would allow us to calm down. (M9‐M)

This non‐reactivity was contrasted with other teachers and school staff. Participants described how other adults got “angry” (M8‐F) and “shouted” (Y2‐F) regularly, which was not felt to contribute to a positive classroom environment. Given the prevalence of negative reinforcement within participants' overall school experiences, positive reinforcement strategies distinguished intervention classes from normal school structures.

Instead, teachers used alternative strategies to manage behaviour. Some strategies were subtle, where teachers could “make the whole class calm without us really noticing” (M7‐M). More overt strategies included the sound from singing bowls, waiting for the class to stop talking, and negotiating for activities that participants enjoyed:

She would say, please stop talking, if you do talk, we will waste more time of our relaxation, and we don't want to waste our time of the relaxation because we need it. She tells us different ways of why we need it. (Y9‐M)

Central to these strategies, instructors modelled the practices they were teaching, which generated mutual respect, which young adolescents also felt differed from other adults in their lives: “She respected us, so people pay that respect back” (M13‐F).

However, when one teacher did not embody the principles that they were teaching and shouted at the class, this negatively impacted on engagement. Shouting undermined the aims of the intervention and damaged the relationship with participants: “I think one time I saw her trying to do a breathing exercise. I don't think it worked” (M24‐M).

#### Agency

Participants reflected on the impact of teachers giving them agency over their choices as a key facilitator of engagement. Adolescents were invited, rather than expected, to take part in activities, and teachers respected their choice to share in the activity or not. As a result, young adolescents felt that teachers respected their “boundaries” (Y13‐M) and enabled them to feel more in control in a new and unfamiliar situation.

An increased sense of agency was especially important for the yoga group. Some participants expressed worries over their flexibility for the various poses, whilst others were self‐conscious over being “judged” (Y11‐M) by peers. In response to these concerns, participants described how teachers created an environment where they had the power to choose:

[Teacher] gave us a choice; if we didn't feel comfortable doing a Yoga pose, we didn't have to do it, but we had to at least try. (Y1‐F)

This approach enabled adolescents to make informed decisions about their engagement and reinforced the positive qualities of their teacher. Such an approach was perceived as creating safe spaces for personal exploration of new experiences within the boundaries of the classroom. This agency over choices was particularly valued by female participants in the yoga group, who noted feeling uncomfortable performing particular poses in mixed‐gender classes: “In front of the boys it's just awkward doing poses like Downward Dog” (Y1‐F).

Despite the positive effects of giving adolescents more agency over their actions, this was also met with some taking “advantage” (M12‐M), creating barriers to engagement as this could lead to disruption, with young adolescents acting up and “messing around” (Y16‐F). This was exacerbated by the large class sizes, as discussed by some participants: “I think the classes would be better if there was less of us in there” (YP5‐M).

In class when she was talking, the class would interrupt her and we had to stop the activity we were doing – like we had to pause a lot. It wasn't really her fault, sometimes the class got a bit over excited and reacted a lot. [The form teacher] had to talk to us about why we should behave. (MP5‐M)

#### Interactivity

Adolescents generally spoke positively about the activities within intervention sessions, which were contrasted with their usual lessons, as they were “physically doing something” (Y7‐M). Subsequently, adolescents viewed intervention classes as more “fun” (M1‐M), facilitating engagement.

For the yoga group, interactive elements aligned with prior expectations of a physical component to classes. Participants enjoyed balancing poses, which were seen as an achievable challenge. Relaxation was a strong theme in both the yoga and mindfulness interventions, where adolescents acknowledged that “in that one hour, it's time to relax” (Y9‐M), which was different to other lessons that had a purely academic focus. Adolescents reflected on how “rare” (Y4‐M) it was that their class was quiet, and they valued the time and space to them to relax: “You shut your eyes and forget about everything.” (Y9‐M).

Despite being classroom‐based, participants also highlighted certain mindfulness lessons as particularly interactive and engaging, predominantly sessions where additional resources illustrated the concepts being taught, such as mindful eating. These positive experiences contrasted with adolescents' initial expectations of mindfulness as “boring”, suggesting that assumptions were not always accurate:

I thought mindfulness was going to be a bit boring, but then when we actually done the lesson, it was better. (M11‐M)

However, sessions with fewer interactive activities were viewed less favourably and acted as a barrier to engagement. Participants felt that some mindfulness lessons were slow and repetitive, which lost their attention. These individuals spoke of their peers in yoga classes with an element of envy:

They had mats and they did yoga we never did. They were doing all this stuff and we were sitting down in our hard chairs. (M20‐M)

### Psychosocial impacts

#### Regulating emotions and calming the mind

Although not all participants experienced positive effects from the interventions, the most commonly reported benefit was an enhanced ability to regulate emotions, which contributed to a greater sense of calm. Breathing exercises were cited as a key mechanism they found helpful for managing stress and anxiety in relation to everyday pressures, including challenging lessons, homework, and exams:

It taught me that if you're in a situation where you feel stress, just do breathing and rest and feel better, then you can get up and fix the problem. (Y10‐M)

One participant recounted how he applied a mindfulness technique to manage a highly stressful incident, which serves as a reminder of the social context of these adolescents’ lives:

Yesterday, something bad happened – there was a stabbing near my house. There were literally five police officers, they blocked off this big main road and then they blocked it off. FOFBOC [feet on floor, bum on chair – grounding exercise] helps me get through stressful things. (M3‐M)

Participants in both groups spoke similarly about the usefulness of breathing, relaxation, and grounding exercises; however, they articulated the mechanisms in distinct ways. Yoga participants described emotional regulation in terms of physical and emotional calm in the face of stressful situations. They used breathing techniques to slow their breathing and heart rate, helping to calm them: “When I'm very angry, I can stay calm and do something called waterfall breath” (Y8‐M).

Participants in the mindfulness intervention described increased emotional control, awareness, and non‐reactivity, where they felt more able to “take control over [their] mind” (M2‐M) and were better able to “control [their] feelings” (M18‐M). They articulated their understanding of the flight or fight response to stress, which helped them to recognize reactions and “take a step back” (M4‐M):

When you're angry you could do anything because you are angry. You can't control yourself, so if you do mindfulness, you can control yourself, you just slowly calm down and relax. (M16‐F)

Adolescents in both groups felt that increased awareness of emotions and strategies to regulate these had decreased conflict within their lives. Instead of reacting, they discussed walking away from arguments, which resulted in “less detentions” (M6‐F) and improvements in their home life and relationships, particularly regarding their families and siblings.

If my brother or sister annoys me, before I used to get angry and annoy them back. Now, I'm a bit calmer so I can navigate it better. (Y1‐F)

#### Positive mindset and confidence

Interventions also supported the development of positive mental states, including a more positive mindset, increased self‐confidence, and shifts in relationships. Participants reflected on how they felt happier, more appreciative, and a “better person” (M8‐F) with this new mindset.

Paying attention to the good things in their lives through conscious redirection of attention to the positive rather than the negative helped young adolescents to achieve this more positive stance. Where participants developed these skills, they realized that “you don't need to carry your bad thoughts with you” (M11‐M), and instead replaced them with more positive ones:

If something bad happens it's easy to take it in and then forget, but before it used to be hard, and it would always be on your mind, even when you were doing something else, but now it's easy. (Y10‐M)

Positivity contributed to young adolescents' well‐being by increasing self‐esteem and self‐confidence. For yoga participants, this increase was strongly tied to performing poses in class, where confidence came from the sense of community generated by everyone doing the same pose together, which reduced self‐consciousness:

I feel like Yoga made me feel a little bit more confident around the school… I hate doing things in front of people, but yoga made me a bit more confident because everyone just does it at the same time. (Y6‐F)

For others, confidence was generated from the trust teachers put in them when they volunteered to lead parts of the class. Standing in front of peers and delivering sequences made yoga participants feel more confident and “accomplished” (Y12‐F). This increase in confidence was also present in mindfulness classes, where adolescents felt more “confident” (M20‐M) and calmer when asked to talk in front of the class. Several participants described their sadness at finishing the programmes, reflecting on their sense of achievement from learning new skills and boost to mood: “it makes me happy” (Y14‐M).

Participants also described various social benefits arising from this newfound confidence. Some felt their existing friendship groups became closer, whilst others felt they had become better people, and had subsequently attracted more friends. Whilst growing social circles were described in positive ways, for a minority of participants, the mental strength to say 'no' to more negative friendship groups was the most important change:

I just thought in a different way. It made me think like shall I be with this friend or this friend, because they might be bad or good. (Y8‐M)

#### Focus and concentration

Intervention sessions were seen as environments where young adolescents could practice the skills of paying attention and focusing, which benefited other classes throughout the curriculum. The majority of participants commented that they could think more clearly after participation, particularly as the interventions progressed. This had direct benefits for attentional focus within sessions, but was also described as translating to other lessons, with young adolescents reporting an increased readiness to learn. Mindfulness participants frequently described their increased ability to control the direction of their attention to continually redirect to the teacher:

We learnt how to control our minds in different places. If you look that way, your mind goes that way, but if you look that way, your mind goes that way. We learnt to look at the teacher, so your mind is focused on the person who is talking. (M20‐M)

For participants in both interventions, there was also a sense that being calmer was more conducive to learning and resulted in less chaotic classrooms. Participants who previously disrupted classes reflected that they didn't talk as frequently during class; consequently, lessons were quieter. Fewer classroom disruptions were advantageous for the whole class, who were better able to focus on the content of academic lessons. For some, mental focus was perceived as beneficial for grades in future tests and exams: “If I get stressed about an exam, my thoughts go everywhere but after Yoga my thoughts… like I have more clear thoughts” (Y1‐F).

Despite benefits for concentration and purposeful directing of attention, some participants felt that ten weeks was not enough to sustain the benefits. For example, one participant reflected that their concentration was better but “I need another eight‐weeks” (Y4‐M).

### Physical performance

The physicality of yoga was appealing to some participants, particularly males. Whilst not as prevalent a theme, participants in the yoga group reflected on benefits to their physical health including strength, energy, stamina, and flexibility. They particularly valued improvements to their athletic performance:

If you do Yoga positions, your legs start to relax, and they don't hurt and it's easier to play [football]. (Y10‐M)

Whilst more salient in the yoga group, some mindfulness participants also reflected that being more present could help manage the pressures of competitive sports:

And it brings you back to that moment and let's say a football match. You're taking a penalty, normally you're going to think about the past or the future. What happens if I miss this penalty? … so that's why you should live in the present. (M20‐M)

## Discussion

This is the first qualitative study to explore the acceptability and impact of separate universal yoga and mindfulness interventions with a large and ethnically diverse sample of young adolescents in the United Kingdom. Initial perceptions of both interventions were varied and could serve as either a barrier or facilitator to initial engagement, though negative expectations were often not borne out in practice. The study identified key factors, some unique to mind–body practices, that impacted intervention engagement and enjoyment. Positive teacher qualities of non‐reactivity and respect, an invitational approach to teaching, and interactive sessions were highly valued by young adolescents and contributed to the development of perceived benefits. Even individuals who did not necessarily enjoy the interventions reported learning tools to help regulate stress and emotions. Indeed, emotional regulation was the most prevalent benefit reported by young adolescents, and they described concrete ways in which they applied the skills learned in their everyday, often stressful lives. These self‐reported self‐regulatory benefits are particularly noteworthy given the increased risk of mental health problems in adolescents from low SES backgrounds.

A key contribution of the current study is the age group of the young adolescent participants (12–13 years old). A recent meta‐analysis highlighted that mindfulness was most effective for older adolescents (aged 15–18 years), but that there were no significant changes for young adolescents (aged 11–14 years; Carsley et al., 2018). Thus, it is particularly important to better understand acceptability, experiences, and any perceived benefits from this population to maximize the potential health‐maximizing effects of mind–body interventions for a wide range of ages. Our findings also contribute to the limited body of work exploring the acceptability of both mindfulness and yoga for adolescents from primarily ethnic minority backgrounds in areas of deprivation. Interestingly, the MYRIAD trial found greater responsiveness to mindfulness training in students identifying as Asian and from schools with higher levels of deprivation, suggesting that mind–body interventions may address unmet needs (Montero‐Marin et al., 2023).

Acceptability of both interventions was linked to young adolescents' expectations of mind–body interventions, which influenced their openness to yoga or mindfulness. Despite negative or indifferent expectations generally developing into more positive experiences, these initial perceptions may present a barrier for young adolescents' engagement. Motivation is strongly influenced by our expectations of enjoyment or success and the value individuals place on a goal (Wigfield & Eccles, 2002), which are important factors in the implementation process (Durlak & DuPre, 2008). This highlights the importance of managing young adolescents' expectations in order to increase acceptability in future school‐based interventions. Interestingly, male participants generally expressed a more favourable view of yoga compared to mindfulness, given the appeal of physical activity. This finding contrasts with established gendered trends in yoga participation, where females are typically more represented than males (Cartwright et al., 2020), suggesting that emphasizing yoga's physical benefits may be an effective strategy to engage young males in health‐promotional interventions.

The current study also identified experiential aspects of intervention classes that were important for young adolescents' engagement, specifically the environment cultivated by the teachers and the invitational nature of mindfulness and yoga. The skill of teachers to remain calm, kind, and non‐reactive was highly valued and respected by young adolescents, which is consistent with previous research on mindfulness and yoga in a school context (Dariotis et al., 2017; Grant, 2017; van Aalderen et al., 2014). Indeed, the qualities modelled by the teachers set the tone for sessions, where adolescents were more likely to emulate the positive behaviour set by teachers. This aligns with the Prosocial Classroom Mediational Model (Jennings & Greenberg, 2009), which points to the importance of teachers embodying the socio‐emotional competencies they teach to foster more positive classroom environments and adolescent outcomes.

Young adolescents also emphasized that acceptability and engagement were strongly linked to intervention content with a strong preference for interactive sessions that provided a contrast to the normal school environment. This was reflected both in pre‐intervention preferences for the more active yoga programme and within intervention content, with variation in enjoyment of the mindfulness sessions, as found previously (McGeechan et al., 2019; Montero‐Marin et al., 2023). Agency over choices was also highlighted as key for facilitating engagement and enjoyment within yoga and mindfulness sessions. When considering adolescence as a challenging period of peer and self‐judgement (Neff & McGehee, 2010), an invitational approach has the potential to empower adolescents. Indeed, Bluth et al. (2016) described an invitational environment as a key factor in the successful implementation of school‐based interventions, and this may be particularly important for universally delivered interventions. Consideration of these factors is important in order to optimize the effectiveness of school‐based well‐being interventions, suggesting the value of co‐designing interventions with children, adolescents, and teachers, whilst recognizing the broader school context (Montero‐Marin et al., 2023). As found in the current study, large class sizes may not provide the optimal environment for mind–body intervention delivery, given the greater potential for disruption.

In addition to describing processes of engagement, the current study identified a range of perceived self‐regulatory, relational, and attentional impacts that were highly relevant for managing young adolescents' current difficulties whilst also establishing a secure foundation for navigating future challenges. These were consistent with the broader mindfulness and yoga evidence base, but evident here with a universal delivery approach in a novel population of young adolescents from a low SES background. Moreover, this study is the first to explore yoga and mindfulness interventions separately within a single study and demonstrate the broad similarities in perceived benefits. Indeed, one of the most frequently cited benefits across the yoga and mindfulness literature, and observed in this study, was improved emotional and stress regulation (Bergen‐Cico et al., 2015; Butzer et al., 2017; Daly et al., 2015; Dariotis, Mirabal‐Beltran, et al., 2016; Sapthiang et al., 2019), with participants also highlighting their enjoyment of relaxation practices to still both mind and body. These self‐regulatory benefits are particularly important given that high levels of self‐regulation are associated with reduced mental health and well‐being problems for adolescents from low SES neighbourhoods (Buckner et al., 2009).

In line with previous research, participants articulated a range of positive changes to mindset following the interventions, including boosts to mood and self‐concept (Cartwright & Doronda, 2023; Case‐Smith et al., 2010; Wang & Hagins, 2015), and self‐confidence (Bhardwaj & Bhardwaj, 2015; Chen & Pauwels, 2014; Dariotis, Cluxton‐Keller, et al., 2016; Dariotis, Mirabal‐Beltran, et al., 2016; Monshat et al., 2013; Vaishnav et al., 2018). Importantly, these changes had tangible social and relational benefits for young adolescents, consistent with previous research (Cartwright & Doronda, 2023; Conboy et al., 2013; Butzer et al., 2017; Van Vliet et al., 2017). This is particularly important given that conflict with siblings is one of the most prominent stressors in adolescents' lives (Dariotis, Cluxton‐Keller, et al., 2016). Moreover, chaos in the home has been identified as a significant risk factor in developing mental health problems for adolescents from low SES neighbourhoods (Devenish et al. 2017). In contrast, strong social bonds have been found to mediate the relationship between deprivation and anti‐social behaviour (Jiang et al., 2020). Therefore, equipping adolescents with the socio‐emotional skills to better manage interpersonal relations and reduce conflict has potential for longer‐term impacts on well‐being in this population.

The increased focus and attentional control described by participants is consistent with previous research (Case‐Smith et al., 2010; Conboy et al., 2013; Costello & Lawler, 2014; Wisner, 2014). This is encouraging given the current study's population, as research has shown that low SES is correlated with reduced cognitive capacity for adolescents (Hackman et al., 2010; Hackman & Farah, 2009; He & Yin, 2016; Sarsour et al., 2011). Lower levels of cognitive abilities and executive functioning in childhood are associated with poorer mental health and lower levels of happiness in later life (Moffitt et al., 2011). Whilst the current study's findings are promising, with observable impacts in the classroom, greater embedding within the curriculum is needed to deliver long‐term benefits.

In adopting a positive psychology lens to view the findings, the importance of fostering environments that respect individual agency, promote compassionate interactions, and nurture strengths across different levels of human experience is consistent with the current findings (Seligman & Csikszentmihalyi, 2000). The compassionate, non‐reactive, and invitational stance of the yoga and mindfulness teachers demonstrates this approach, as it created a space where students felt both respected and empowered. This approach aligns with the principles of 'positive institutions' in which compassionate education creates supportive environments that enable growth and enhance well‐being (Neff & Germer, 2013; Waddington & Bonaparte, 2024). From a biopsychosocial perspective, the interventions in the current study promoted health and well‐being at the subjective, individual, and group levels, aligning with contemporary perspectives on well‐being that emphasize mindfulness and self‐compassion as multifaceted health‐promoting practices (Baer, 2003). On the *subjective* level, adolescents' sense of respect and agency seems to have fostered positive emotional states which helped them regulate stress. At the *individual* level, adolescents showed increased self‐compassion and resilience, key traits in positive psychology that help individuals thrive amid adversity (Neff, 2003). At the *group* level, the relational foundation of the intervention helped foster a sense of community and mutual support between young adolescents and teachers, illustrating how positive psychological interventions have the potential to benefit both individual well‐being and social connection and cohesion (Fredrickson, 2001).

The concept of social cohesion further emphasizes the value of universal and whole‐school approaches to support well‐being. Whilst previous reviews have concluded that targeted mental health and well‐being support may be more effective (Sanchez et al., 2018; Werner‐Seidler et al., 2017), a growing evidence base points to the value of universal approaches (Clarke et al., 2021; Durlak et al., 2011; Mackenzie & Williams, 2018; O'Connor et al., 2018). Importantly, universal approaches that incorporate all individuals may be more effective in reducing mental health burden, whilst also promoting flourishing across the full spectrum of children and adolescents (Greenberg & Abenavoli, 2017). Universal implementation of yoga and mindfulness within schools thus has the potential to reach large numbers of children and adolescents, equipping them with the tools to better cope with challenges in their everyday lives. Supporting children and adolescents to develop socio‐emotional skills is more important than ever with the exacerbation of mental health problems following the COVID‐19 pandemic (Newlove‐Delgado et al., 2021), particularly for those from disadvantaged backgrounds (Pearcey et al., 2020).

Whilst our findings are promising in relation to the acceptability and impact of mindfulness and yoga for children and adolescents from areas of high deprivation, mind–body interventions are not a panacea or a “quick fix” (Broderick & Frank, 2014 p. 42) for mental health and well‐being problems. It is encouraging that participants articulated benefits and socio‐emotional skills after only 10 weeks; however, regular practice for an extended period of time is important for meaningful and optimal benefits (Durlak et al., 2011; Sherman, 2012). Indeed, our wider study found short‐term benefits following classes, but no significant changes were found on measures of well‐being (Sumner et al., in prep). Whilst universal interventions have the potential to increase psychological flourishing across a wide spectrum of individuals, without further reinforcement of the socio‐emotional skills developed, they may lack the intensity to effect measurable change (Greenberg & Abenavoli, 2017). The fact that our participants spoke about how they did not want the programmes to end suggests that longer‐term interventions would be beneficial within a school setting. However, there are practical barriers to this in schools (Stattin & Kerr, 2009) that would require embedding of interventions in the school day with increased buy‐in from senior management and sufficient resources (Wilde et al., 2019). To this end, the Department for Education (2021) has championed School Mental Health Leads, who are empowered to develop and oversee a whole‐school approach to well‐being within their setting, highlighting a national commitment to promoting mental health and well‐being in schools.

Moreover, any potential benefits of universally delivered mind–body interventions need to be balanced against any potential harms. The recent MYRIAD trial found some evidence of poorer outcomes in adolescents' well‐being and emotional affect in the mindfulness group (Kuyken et al., 2022), consistent with the adult literature (Dobkin et al., 2012; Kerr et al., 2011; Lomas et al., 2015). Yoga and mindfulness interventions increase self‐awareness, which could, in turn, lead to increased awareness of negative emotions and stressors (White, 2012). A nuanced understanding of these findings is required within the context of universal delivery, so children and adolescents may need additional guidance through this process, particularly a low SES population who may be more exposed to stressful life events (Kim et al., 2013) as evidenced in the current study.

### Strengths and limitations

A notable strength of this in‐depth qualitative study was the inclusion of a substantially larger and more diverse sample, compared to previous qualitative research (Bannirchelvam et al., 2017; Bluth et al., 2016; Butzer et al., 2017; Case‐Smith et al., 2010; Dariotis, Cluxton‐Keller, et al., 2016; Dariotis, Mirabal‐Beltran, et al., 2016; McGeechan et al., 2019). This allowed for a wide range of experiences and impacts. Uniquely, the current sample predominantly consisted of young adolescents from Black, Asian, and Minority Ethnic backgrounds. Thus, the study accessed groups that are often underrepresented in research, giving voice to a more diverse group of young adolescents.

Whilst the findings may have application for other urban schools in neighbourhoods with high deprivation, the findings are limited in a number of ways. Firstly, they were based on a single London secondary school and therefore the findings should be interpreted with caution. Secondly, purposeful sampling through schoolteachers to ensure variation in experiences may have led to bias towards those with more positive experiences, although this was not evident in the narratives where participants openly shared more critical insights. Member checking, allowing the participants to review and reconfirm the accuracy of the findings, may be a useful strategy to increase the trustworthiness of the data in future studies. Lastly, the timing of the interviews within a week of the interventions ending facilitated accurate recall (Sapthiang et al., 2019), but does not reveal the longer‐term impact on socio‐emotional skills. Future research should adopt a longer post‐intervention time period to explore the sustainability of perceived benefits.

## Conclusion

Our study expands the current evidence base regarding the acceptability and impact of yoga and mindfulness interventions, with a novel population of young adolescents from a low SES area in the United Kingdom, engaging in universally delivered mind–body interventions within the school curriculum. The research has highlighted a range of benefits for young adolescents' socio‐emotional, attentional, and relational skills, contributing to improved well‐being and flourishing. Moreover, it demonstrated that mind–body interventions were most acceptable to young adolescents when they were delivered by a teacher with positive and non‐reactive qualities, who facilitated an invitational environment, with engaging, interactive class‐based activities, highlighting the importance of compassion and respect in creating supportive environments that enable growth and enhance young adolescents' well‐being. The findings will assist schools in finding practical solutions to promote and support the well‐being of children and adolescents, which have become even more imperative in the context of increasing mental health problems exacerbated by the COVID‐19 pandemic.

## AUTHOR CONTRIBUTIONS

**Amy L. Sumner:** Conceptualization; investigation; methodology; formal analysis; data curation; writing – original draft; writing – review and editing; project administration. **Tina Cartwright:** Conceptualization; methodology; data curation; writing – original draft; writing – review and editing; supervision; funding acquisition; visualization; formal analysis. **Haiko Ballieux:** Conceptualization; methodology; supervision; funding acquisition; writing – review and editing; visualization. **Trudi Edginton:** Conceptualization; methodology; writing – review and editing; visualization; supervision; funding acquisition.

## Supporting information


Data S1:

## Data Availability

The data are unavailable because the institutional ethical approval for working with children did not include consent for data sharing. A comprehensive table featuring anonymised quotes has been included in the Data S1 to further evidence the themes identified in the current study.

## Footnotes

^1^
Quotes are referenced by intervention group (Yoga/Mindfulness), assigned participant number (1–24), and gender (Male/Female) e.g. M9‐M participated in the mindfulness intervention and identified as male.
