# Supplementary material for: School‐based yoga and mindfulness interventions for young adolescents: A qualitative study in a disadvantaged area
Source: Br J Health Psychol. 2025 Mar 18;30(2):e12793. doi: 10.1111/bjhp.12793 (PMC11920380; doi:10.1111/bjhp.12793)
Supplement: Supplementary file 1 — Data S1: [file BJHP-30-0-s001.docx]

**Supplementary Material 1: Interview topic guide**

**Section One: Background Information**

- How old are you?
- How long have you been at this school? What’s it like?
- Do you have a favourite subject/teacher?

**Section Two: Perceptions of Yoga/Mindfulness**

- Before your classes last term, what did you think yoga/mindfulness would be like?
  - Did you have any experience of yoga/mindfulness before these classes?
- How did you feel when your teacher told you that you would be doing yoga/ mindfulness last term?
- What, if anything, did you want to get from the classes?

**Section Three: Experience of Yoga/Mindfulness Classes**

- How did you find the yoga/mindfulness classes?
  - What did you like most about the classes?
  - Was there anything you didn’t like?
- What was it like doing it with your whole class?
- How did you find the teacher?
  - What did you like most about the teacher?
  - Was there anything about them that you didn’t like?
- Did you do any practice in between classes?
  - Homework?
  - At school?
- How do you feel now these classes have come to an end?
- Have you continued any of the practices you learned?

**Section Four: Perceived Impact of Yoga/Mindfulness**

- How did you feel when you were doing yoga/mindfulness in class?
- Did you feel any different before/after the class?
- What changes, if any, have you noticed in yourself since doing yoga/mindfulness?
  - Physical health: pains, flexibility, energy, sleep?
  - Mental health: Stress, anxiety, anger, depression?
  - How you feel about yourself: self-acceptance, esteem, understanding?
- What changes, if any have you noticed in your life at school since doing yoga/ mindfulness?
  - Classroom behaviour?
  - Attention in class?
  - Views of school?
- What changes, if any have you noticed in your life at home since doing yoga/ mindfulness?
  - Relationships with family?
  - Overall atmosphere?
- Have you noticed any changes in your friendships since doing yoga/mindfulness?
- What difficulties, if any, have you experienced related to yoga/mindfulness practice?

**Section Five: Overall Comments**

- Overall, how did you feel about your school introducing yoga/mindfulness into your PSHE classes?
- To what extent would you recommend yoga/mindfulness to other pupils/schools?
- What could be changed or improved about the classes in the future?
- Is there anything else you’d like to add about your experience of yoga/mindfulness at school?

**Supplementary Material 2: Yoga4Schools intervention**

Overview of Yoga4Schools curriculum

| **Week** | **Theme** | **Aims and objectives** |
| --- | --- | --- |
| 1 | Introduction to Yoga: Taking the first step | - To introduce yoga and the concept of the ‘beginners mind’ as a mind set to approach new experiences (e.g. yoga classes) without set expectations |
| 2 | Being present | - To introduce the concept of awareness and being present in the current moment without judgement – with links to mind-body awareness |
| 3 | Stress management | - To notice feelings of stress - To promote an awareness of responses to stress and ways to manage these through yoga |
| 4 | Embracing challenges | - Following on from stress, this lesson will explore how students can manage other difficult emotions with a growth mind set and encourage them to change their perspective on difficult situations |
| 5 | Energy and performance | - To re-energise pupils with empowering asana, focusing on using yoga to support performance |
| 6 | Sleep | - To increase understanding on how yoga can contribute to better and improved sleep - To give pupils strategies to improve their sleep hygiene |
| 7 | Mental focus and concentration | - To focus on balance to improve focus and concentration - To teach pupils techniques to increase their concentration with reference to benefits both inside and outside of school. |
| 8 | Being your best self | - To encourage pupils to be true to themselves, be compassionate to their imperfections and stop comparing themselves to others - To aim to increase their resilience and belief in themselves to achieve. |
| 9 | Expressing gratitude | - To encourage pupils to reflect on the world, their relationships, and themselves to explore what they feel grateful for and how they can express their gratitude through acts of kindness to the world and other people |
| 10 | Yoga in everyday life | - To summarise the most helpful ideas from the last nine weeks and encourage pupils to think about ways they can continue their yoga practice. |

Full programme freely available DOI: [10.13140/RG.2.2.21668.19841](http://dx.doi.org/10.13140/RG.2.2.21668.19841)

**Supplementary Material 3: Mindfulness .b intervention**

Overview of .b mindfulness curriculum.

| **Week** | **Theme** | **Aims and objectives** |
| --- | --- | --- |
| 1 | Introduction to mindfulness | - An introductory lesson persuades adolescents that mindfulness is worth learning about by making it relevant to their lives in an engaging and entertaining way. |
| 2 | Playing attention | - To introduce pupils to the faculty of their attention and how they can purposefully direct attention using simple tools - To introduce key attitudes to attention-training: kindness, patience, repetition |
| 3 | Taming the animal mind | - To explore that the mind has a life of its own - To nurture an attitude of curiosity, kindness, acceptance and openness that helps to deal more skilfully with these fluctuating mind-states |
| 4 | Recognising worry | - To develop understanding that the mind habitually interprets and ‘tells stories’ about what is happening – we can get stuck in our heads and ‘ruminate’ or ‘catastrophise’ - Provide tools and practices to manage these experiences – switching from ‘thinking’ mode to ‘sensing’ mode |
| 5 | Being here now | - To explain how ‘autopilot’ prevents us from being alive and awake to our experience in the here and now - To learn to appreciate and savour the pleasant - To learn how to respond rather than react to the unpleasant |
| 6 | Moving mindfully | - To develop understanding that Mindfulness is also about movement. We spend time doing actions ‘mindlessly’ (e.g. walking) - Learning to move mindfully can also be used as a resource for peak performance in sport, music, and the performing arts |
| 7 | Stepping back | - Understand that we have the capacity to ‘step back’ from thoughts - Identify some of the particular ‘thought-buses’ that pass through your mind and recognise that you don’t have to ‘get on the bus’ of these difficult thoughts |
| 8 | Befriending the difficult | - Understand stress: where it comes from, why it is necessary, how it works and the potentially harmful effects. - Learn to respond rather than react, by ‘turning towards’ and ‘being with’ difficult emotions. |
| 9 | Taking in the good | - Encourage an appreciation of what is good in life and explain the ordinary can be experienced as ‘good’ if we are fully aware of it |
| 10 | Pulling it all together | - To identify what they have found most useful and consider in what areas of their life they might apply their new mindfulness skills. |

For further information about the programme and training:  <https://mindfulnessinschools.org/school-mindfulness-lead/>

**Supplementary Material 4: Illustrative quote table for themes and sub-themes.**

| **Theme** | **Sub-theme** | **Yoga Intervention Group** | **Mindfulness Intervention Group** |
| --- | --- | --- | --- |
| **Facilitators and Barriers to Engagement** | **Expectations** | “I didn’t really know, I just knew the stuff you see in films and yeah, like the stereotypical thoughts, but I didn’t really know what we were going to do.” (Y1-F)  “You can help… your tensions in your body, so like some boxers like [Name] use it, so I don’t really mind doing it.” (Y3-M)  “I wanted to do it because I felt like it was going to make me more flexible and stronger” (Y4-M).  “I was excited because we’ve never done anything like Yoga before and it’s like something new to the school.” (Y7-F)  “When we were first told, I thought it was going to be like… I thought Yoga was we were going to meditate and stuff.” (Y17-M) | “At first, I had no idea I don’t understand what mindfulness is. I know what Yoga is, I’m not too keen on it, but mindfulness I don’t know what it is. I thought I could give it a try, hopefully it won’t be too bad.” (M1-M)  “I didn’t have the best expectations, because Mindfulness kind of sounds boring. That’s what I thought, that was my assumption.” (M9-M)  “It was quite weird because we were learning to train our minds which we have never really done in school before, but I was excited to find out what it was.” (M8-F)  “I knew it was something about like calming people down, I knew it was something like that but I didn’t know fully.” (M10-F) |
|  | **Teacher qualities** | “She was like really calm, she didn’t raise her voice that much, that’s why most of the class listened to her, because she didn’t shout at us a lot. She just waited for us to calm down and sort of because we needed to calm down, because we were doing Yoga, she knew how to help us calm down.” (Y1-F)  “Say we would lose our focus, [yoga teacher] would let us go into the Childs pose and calm ourselves and then go back to it. With our tutor, he would be there giving out detentions.” (Y6-F)  “She was calm…she knew how to control the children.” (Y7-F)  “She was nice because she would care about everyone, and she would go around everyone be like are you okay? Do you need rest time? She was just a nice teacher overall.” (Y8-M)  “If she wants to say something… she doesn’t really say anything negative, but if she did need to say something negative, she would say it in a relaxing way, which would make us relaxed. Teachers in the school like, they would shout at us.” (Y9-M)  “She would never like get angry too easily unless someone did something, like consistently talks.” (Y13-M) | “She wouldn’t get annoyed that we were asking questions; she will take the time to explain it. Even though, sometimes the kids in our class they misbehaved, she still stayed calm.” (M2-M)  “She never really got frustrated, she like understood we are kids and like that is going to happen. She was calm and quite positive, even though we might have made her frustrated, she never really showed that, just happiness.” (M7-M)  “Yeah, some teachers, when they get angry, they get so angry and the class gets angry at them. She is very calm; she doesn’t do anything to make the kids not like her.” (M8-F)  “She respected us, so people pay that respect back.” (M13-F).  “She was nice, in general. Like her teaching, she wouldn’t shout, and she barely ever shouted. I’m not going to lie, our class, there was a time when our teacher didn’t come and so the class wasn’t being very nice and she didn’t really shout but she just said should I just leave and when she stopped everyone went quiet and listened to her for the rest of the lesson.” (M14-M)  “Most teachers would like shout to get us silent, but she would calmly say stop talking or she would ring the bell and that would stop us from talking.” (M18-M) |
|  | **Agency** | “It was like Downward Dog, you had to go forward and in front of the boys it’s just awkward doing poses like that.” (Y1-F)  “Sometimes, whilst I’m doing it, Miss lets me sit on the chair for like five minutes and then when I’m ready I get to go back.” (Y4-M)  “If we were tired or anything we could rest for a bit and then go back to it – she wasn’t pushing us.” (Y11-M)  “She respected our boundaries of where we can go and where we can’t.” (Y13-M)  “She did a lot of balances and told us in detail what to do, so we wouldn’t hurt ourselves or endanger ourselves or around us.” (Y14-M)  “Sometimes we can have time to do what we like.” (Y21-M) | “It’s totally up to you what you do.” (M1-M)  “She wasn’t strict, like you didn’t have to do it if you didn’t want to.” (M3-M)  “[mindfulness classes] were more free…we could choose.” (M16-F)  “She would try to encourage us but would never force us” (M19-F). |
|  | **Interactivity** | “The balancing, it just felt a bit more relaxing than Downward Dog, it also like made me feel like… I don’t know I just liked doing it more than the others.” (Y1-F)  “We would lie down sometimes, lie down and close our eyes…it was calming; well relaxing.” (Y4-M)  “The best part was the sun salutations and the upside-down dog.” (Y5-M)  “I like yoga because you are actually physically doing something, and you can relax yourself.” (Y7-F)  “It’s not all about lessons, lessons, lessons; in that one hour, it’s time to relax.” (Y9-M)  “I learnt new things and the thing I like. I learnt how to do lots of different poses.” (Y14-M)  “I liked that we could communicate. Normally we would just be writing there, but we were able to communicate and learn from each other. She also helped us to give positive advice to each other.” (Y16-F)  “I liked the relaxing bit at the end; it was the most relaxing of the whole class…and it’s still better than PSHE.” (Y17-M) | “It was fun because we don’t get to do that in [other] classes.” (M1-M)  “Sometimes it was a bit boring but when we were doing activities, then the whole class was alive.” (M7-M)  “We did lots of activities. We did loads of demonstrations and the mindful eating was really fun, everyone liked that!” (M8-F)  “[It was] relaxed and it was very quiet, because our class is never quiet, we are always talking. This was the first time in like the whole year that we were quiet, it was very relaxing.” (M8-F)  “The lessons are nice, and we get…we are allowed a lot of interactive activities and stuff.” (M9-M)  “[My favourite lesson was] probably the walking one because usually we just stay inside the classroom. We got to see everyone; it was like a tour.” (M16-F)  “I think you should do mindfulness in other schools but add other activities and make it more fun for the person and then they will like it more and they will learn from it.” (M23-F) |
| **Psychosocial impacts** | **Regulating emotions and calming the mind** | “If my brother or sister annoys me, before I used to get angry and annoy them back. Now, I’m a bit calmer so I can navigate it better.” (Y1-F)  “You need yoga in your life to relax.” (Y2-F)  “I think it helps calm down your mind from stress.” (Y3-M)  I think since it [yoga] has started, I have relaxed more. When I get home from school and I’m really tired, I can just lie down, close my eyes and do the breathing….I feel more relaxed when my brain is calmed down.” (Y4-M)  ‘I’ve stopped being so angry…sometimes.” (Y7-F)  “Whenever you are angry or stressed, maybe if you get too angry, you can do a yoga position and it will make you calmer.” (Y8-M)  “It taught me that if you’re in a situation where you feel stress, just do breathing and rest and feel better, then you can get up and fix the problem.” (Y10-M)  “When I’m angry I can calm myself down.” (P11-M)  “I find it relaxing because if it’s a test or something, I can control my breathing and it would relax me.” (Y14-M) | “A benefit for me is that I can deal with stress better, like before I used to not talk to people. Now, I’ll understand what’s happening and see if there is anything I can do and acknowledge things that I can’t do, that are outside my control, and accept it.” (M2-M)  “Mindfulness helps me to not react to it and most of the time I just take a step back before doing anything.” (M4-M)  “It’s the only thing that calms me down.” (M8-F)  “[Breathing exercises] calmed me and made me feel relieved, I don’t know how to explain it. It was a good feeling.” (M10-F)  “Mindfulness teaches you don’t react because they know they are getting to you; don’t react and take a different approach, like ignore them.” (M12-M)  “You don’t need to be stressed by something, as long as you just stop and count your breathing and control your breathing.” (M15-F)  “[I can] control [my] feelings, instead of the feelings controlling [me].” (M18-M).  “I didn’t like the subject, but I learnt stuff from it. So, I learnt your emotions, how to control your emotions, your mind, how to deal with anger, stress, you know depression. You know how to like, because the mind makes up a lot of fake stories and you have learned how to throw it away and be happy.” (M5-M) |
|  | **Positive mindset and confidence** | “The tension in your muscles was just gone.” (Y1-F)  “[Me and my friends] has something in common, to talk about and do together.” (Y1-F)  “It helps you with your core strength.” (Y4-M)  “I’m happy, before Yoga, I just used to be quiet, at home. I wasn’t a social guy, after the first few weeks, I used to start talking to people more. I just got more confident.” (Y5-M)  “I feel more positive about the week ahead.” (Y6-F)  “It was probably just that all the people were doing the poses at the same time and no one was doing it by themselves. So, we would be doing it with other people, and no one would be looking at you, and that got me a bit more confident.” (Y6-F)  “It makes you more aware, it makes you get in less arguments with your siblings. Be better…be nicer and kinder.” (Y10-M)  “It made me feel calmer and more confident. It made me a less nervous person.”(Y12-M)  “I think my confidence rose…and I can do speaking in front of the class without that much hesitation.” (Y13-M) | “It helps your life. You try to think just good things, for example before mindfulness, I always think bad things but after the mindfulness, I try to think good things.” (M5-M)  “It made me feel a bit happier and more appreciative of the things I have. It made me realise that I am in a situation where I should be happy and not upset and it made me think positively.” (M7-M)  “I feel that me and my friends have got closer because, say if one person gets angry, we know how to calm them down, so it brings us together.” (M10-F)  “I see the world differently.” (M12-M).  “It’s kind of made me more grown up I suppose.” (M13-F)  “When you’re not stressed, you make like good choices. Like imagine someone is doing something as a group and they tell you to join but then you don’t join them because you know what they are doing is bad.” (M17-M)  “I learnt a lot of stuff from mindfulness, I got a lot of benefits from it. Like now I feel more confident in what I’m doing. I don’t think I would have been in the past.” (M20-M) |
|  | **Focus and concentration** | “It makes you feel relaxed, so for the rest of the week we weren’t tired, just more relaxed and like it helped us stay awake in lessons and focus more.” (Y1-F)  “I’m finding my concentration, it’s a bit better now.” (Y3-M)  “It makes you better behaved in class, but it’s the rest of the class as well.”(Y8-M)  “After lunchtime everyone is excited and happy. We run into class and we jump up and down and the teacher calms us down. But after yoga, we have tutor time, we walk calmly, we sit down calmly.” (Y9-M)  “It’s because it’s a double lesson, so you have double of the lesson, like Yoga lesson after would make you less tired or concentrated on the work. It’s like 100 minutes is a lot for one lesson. So, having Yoga would be better for your concentration on your lessons.” (P20-F) | “In lessons, I kind of talk a lot. It’s helped me to calm down and not talk that much. I think it was the thought buses because I can be looking at the teacher but talking about what happened on Saturday or something like that. I just go off task; it helped me to stay focused.” (M10-F)  “Usually in our class when the teacher tells us to stop talking, no one really listens, and everyone ends up getting detentions and stuff. Since mindfulness has been here, when the teacher tells us to stop, we stop and don’t get into trouble.” (M18-M)  “I come into lessons usually like excited and jumping up and down and loud. When we get into lessons now, we are quieter. We are more relaxed and less chaotic.” (M19-F) |
|  | **Physical performance** | “I think I’m a bit more flexible now, before I used to be very inflexible.” (Y3-M)  “Yeah, it helps out for more stamina. I play full minutes, like I play like full ninety minutes and I don’t get tired.” (Y3-M)  “She gave us advise on if you play sport, football, you should do this position.” (Y8-M)  “It makes me run faster. Because if you have stiff legs, its harder to run. If you loosen up your legs and loosen up your body, it makes you feel free.” (Y9-M)  “I think if we did more movement in the lesson, it makes us more energetic.” (Y13-M)  “It prevents any like body aches, because Yoga stretches your muscles out and warms you up for the day and relaxes you and relieves stress.” (Y14-M) | “Maybe it can help when you want to get better at a certain sport.” (M16-F)  “I did think that the breathing thing actually worked because I had a really important football game, I tried to do that thing before the game, it made me even more nervous.” (M24-M) |
